# A Changing Light Environment Induces Significant Lateral CO_2_ Diffusion within Maize Leaves

**DOI:** 10.3390/ijms232314530

**Published:** 2022-11-22

**Authors:** Han-Yu Wu, Qing-Qing Zou, Wen-Tao Ji, Ying-Wei Wang, Wang-Feng Zhang, Chuang-Dao Jiang

**Affiliations:** 1 Key Laboratory of Plant Resources, Institute of Botany, Chinese Academy of Sciences, Beijing 100093, China; 2 Key Laboratory of Oasis Eco-Agriculture, Xinjiang Production and Construction Corps/College of Agronomy, Shihezi University, Shihezi 832003, China

**Keywords:** fluctuating light, photosynthesis, respiration, CO_2_ partial pressure, leaf structure, sorghum

## Abstract

A leaf structure with high porosity is beneficial for lateral CO_2_ diffusion inside the leaves. However, the leaf structure of maize is compact, and it has long been considered that lateral CO_2_ diffusion is restricted. Moreover, lateral CO_2_ diffusion is closely related to CO_2_ pressure differences (ΔCO_2_). Therefore, we speculated that enlarging the ΔCO_2_ between the adjacent regions inside maize leaves may result in lateral diffusion when the diffusion resistance is kept constant. Thus, the leaf structure and gas exchange of maize (C_4_), cotton (C_3_), and other species were explored. The results showed that maize and sorghum leaves had a lower mesophyll porosity than cotton and cucumber leaves. Similar to cotton, the local photosynthetic induction resulted in an increase in the ΔCO_2_ between the local illuminated and the adjacent unilluminated regions, which significantly reduced the respiration rate of the adjacent unilluminated region. Further analysis showed that when the adjacent region in the maize leaves was maintained under a steady high light, the photosynthesis induction in the local regions not only gradually reduced the ΔCO_2_ between them but also progressively increased the steady photosynthetic rate in the adjacent region. Under field conditions, the ΔCO_2_, respiration, and photosynthetic rate of the adjacent region were also markedly changed by fluctuating light in local regions in the maize leaves. Consequently, we proposed that enlarging the ΔCO_2_ between the adjacent regions inside the maize leaves results in the lateral CO_2_ diffusion and supports photosynthesis in adjacent regions to a certain extent under fluctuating light.

## 1. Introduction

It is well known that the CO_2_ required for photosynthesis mainly enters the leaves through the stomata. Photosynthesis depends on the diffusion of CO_2_ to carboxylation sites through gas and liquid phase pathways in the leaves. Gaseous diffusion refers to the transport of CO_2_ in the atmosphere through the substomatal cavity and the intercellular airspaces [1]. Thereafter, the diffusion of CO_2_ in the intercellular airspaces to the carboxylation site mainly depends on the liquid pathway. Generally, the CO_2_ in the intercellular airspaces is first dissolved in water and gradually diffuses to the carboxylation site in mesophyll cells. Diffusion pathways include the cell wall, plasma membrane, cytoplasm, chloroplast envelope, and matrix [2,3,4,5]. As the rate of gaseous diffusion is much higher than that of liquid diffusion, maintaining a relatively high gaseous diffusion in plant leaves plays a very important role in enhancing photosynthetic efficiency [6,7,8].

Photosynthesis in the leaves mainly takes place in the palisade tissue. The palisade tissue is located on the adaxial side of the leaves and contributes to the leaf structure. In order to reduce leaf transpiration and maintain leaf water balance, the stomata of many plants are mainly distributed on the abaxial side of the leaves or on both sides of leaves, with more on the abaxial side. In most cases, CO_2_ entering the leaf interior from the stomata needs to gradually diffuse to the carboxylation site of the palisade cells in the vertical direction of the leaf blades [9]. Therefore, the gaseous diffusion of CO_2_ in the vertical direction of the leaves plays an important role in photosynthesis. In addition to transverse diffusion (vertical direction), some studies have demonstrated that there is obvious lateral CO_2_ diffusion in many plant species through gas exchange, and the lateral diffusion of CO_2_ can support photosynthesis in adjacent regions [10,11,12]. In fact, for both the transverse and lateral diffusion of CO_2_ in the leaves, it is generally considered that gaseous diffusion depends on the leaf porosity. Plants with low leaf porosity have low transverse and lateral gaseous diffusion; on the contrary, plants with high porosity have high gaseous diffusion [7,11,13]. Previously, it was considered that the leaf intercellular airspace (IAS) was the key factor determining porosity and that it affected CO_2_ gaseous diffusion within the leaves. Hence, the concept of gaseous conductance was developed based on the IAS and porosity [14,15,16]. Recently, it was thought that tortuosity in the leaf IAS and lateral path lengthening of CO_2_ diffusion are also important factors affecting lateral diffusion. Accordingly, Earles et al. [7] corrected the calculation of gas conductance by considering the tortuosity and lateral path lengthening in order to measure and analyze gaseous diffusion more accurately. Furthermore, there is evidence that CO_2_ gaseous diffusion will also be influenced by IAS connectivity [7,17]. Consequently, lateral gaseous diffusion depends on porosity and related characteristics.

In previous studies, it was demonstrated that although the mesophyll cells of many plant species, such as soybean, were loosely packed into a given leaf volume and had higher porosity, the lateral diffusion of CO_2_ was still very low [11,18]. It was suggested that these plant species have a vascular bundle sheath extension (BSE) structure. BSEs can lead to the regionalization of photosynthetic function by compartmentalizing the leaf into small areoles, thereby restricting lateral CO_2_ diffusion [18]. This type of leaf with a vascular BSE structure is called a heterobaric leaf. On the contrary, homobaric leaves, in which such BSE structures are absent, apparently allow for significant lateral diffusion of CO_2_ within the leaves. However, lateral CO_2_ gaseous diffusion can occur in heterobaric leaves when a larger ΔCO_2_ is induced between the adjacent regions [19,20]. Accordingly, BSE, as a physical barrier in heterobaric leaves, may no longer be the limiting factor affecting lateral CO_2_ diffusion under a larger ΔCO_2_.

As an important C_4_ plant, maize has stomata on both sides of the leaf, the mesophyll cells are densely arranged, and the porosity is very low. Photosynthesis can be carried out effectively on both the adaxial and abaxial sides of the leaf. To date, many studies have proposed that CO_2_ gaseous diffusion within maize leaves is very low and is not only in the vertical direction [21] but also in the lateral diffusion [19,20]. Although the mesophyll cells of maize are densely arranged and have a lower porosity, based on studies of heterobaric leaves, we speculated that when the ΔCO_2_ between the adjacent regions of maize leaves is large enough, it may also overcome the resistance of gaseous diffusion and result in lateral diffusion, thereby affecting the photosynthesis of adjacent regions. Light is an important environmental factor affecting the photosynthesis, growth, and development of plants. Light fluctuations, including heteronomous or patchy light environments, may occur more frequently under field conditions [22,23]. Highly heteronomous or patchy light environments between adjacent leaf regions under natural conditions are likely to produce a significant ΔCO_2_ and lateral diffusion inside the leaves. To test this hypothesis, the leaf structure and gas exchange of maize, sorghum, and cotton were analyzed herein. Under complex light environments, potential lateral CO_2_ diffusion inside the leaves may be of great significance for maintaining carbon assimilation and improving water use efficiency.

## 2. Results

### 2.1. Differences in the Leaf Structure of Various Plant Species

**Figure 1:**
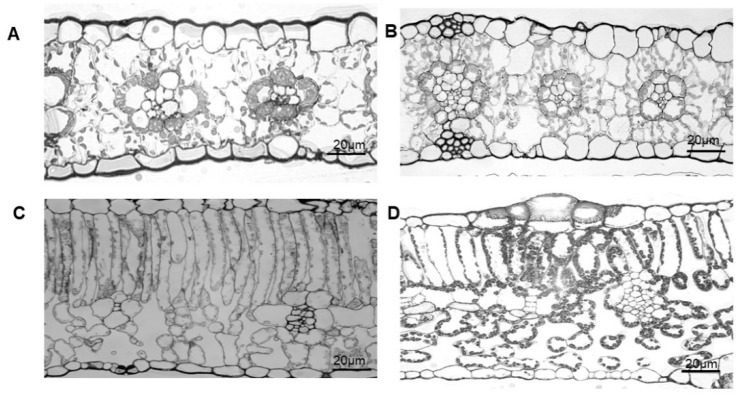
Leaf microstructure of various plant species. (**A**) maize; (**B**) sorghum; (**C**) cotton; (**D**) cucumber.

**Figure 2:**
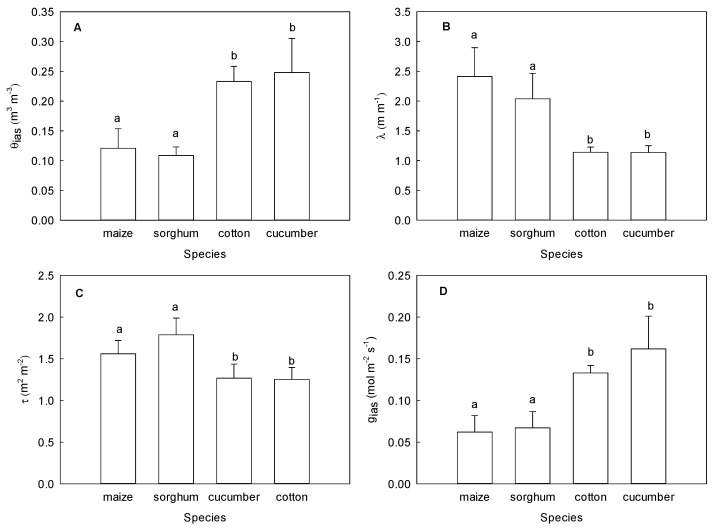
The differences in mesophyll porosity (θ_ias_), lateral path lengthening (λ), tortuosity factor (τ), and gaseous conductance of the intercellular airspace (g_ias_) in maize, sorghum, cucumber, and cotton. (**A**) mesophyll porosity; (**B**) lateral path lengthening; (**C**) tortuosity factor; (**D**) gaseous conductance of the intercellular airspace. Data are means ± standard error (n ≥ 3); different lowercase letters indicate significant differences between different species at the *p* < 0.05 level.

As shown in Figure 1A,B, there was no differentiation between the palisade and sponge tissues in the mesophyll tissues of the maize and sorghum leaves. The mesophyll cells were more densely packed into a given leaf volume and had a typical rosette morphology. However, there was obvious differentiation of the palisade and sponge tissues in the mesophyll tissues of the cotton and cucumber leaves. Moreover, the cells of the palisade tissue were densely arranged, and the cells in the sponge tissue were loosely arranged (Figure 1C,D). It was noticed that the cell arrangement of the cotton and cucumber leaves was looser than that of maize and sorghum in terms of the microstructure. Further statistics showed that the value of mesophyll porosity (θ_ias_) was 60% lower in maize and sorghum than in cotton and cucumber, while the lateral path lengthening (λ) and tortuosity factor (τ) were higher in maize and sorghum than in cotton and cucumber (Figure 2). Accordingly, the conductance of the intercellular airspace (g_ias_) was significantly lower in the leaves of maize and sorghum than in cotton and cucumber.

### 2.2. Effect of Local Illumination on the Respiration Rate of Adjacent Region in the Same Leaves

**Figure 3:**
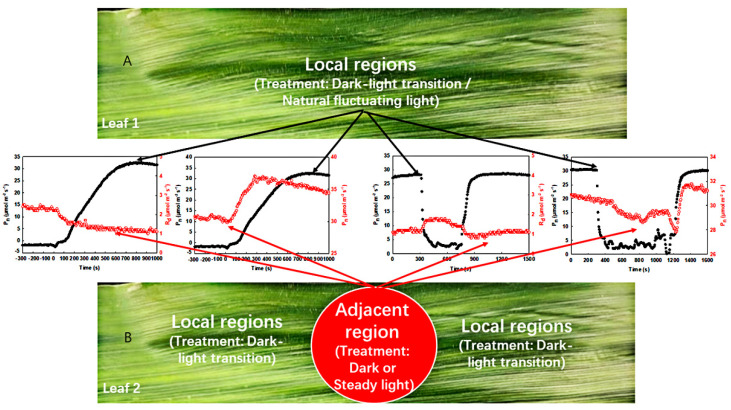
Schematic of gas exchange measurement. (**A**) Photosynthetic induction under the dark–light transition or photosynthetic rate under natural fluctuating light in the local regions; (**B**) respiration in darkness or photosynthetic rate under steady light intensity in the adjacent region; dark–light transition, D–L transition; steady light treatment, SL treatment.

In this study, the area of the adjacent region was only 2.5 cm^2^, while the area of the local regions was at least 100 times larger than that of the former (Figure 3). Clearly, the adjacent region had little influence on the gas exchange in the local regions, while the local regions had a great impact on the former. Thus, more attention has been paid to the changes in respiration and photosynthetic rate in the adjacent region when the light environment of local regions changes artificially or naturally.

**Figure 4:**
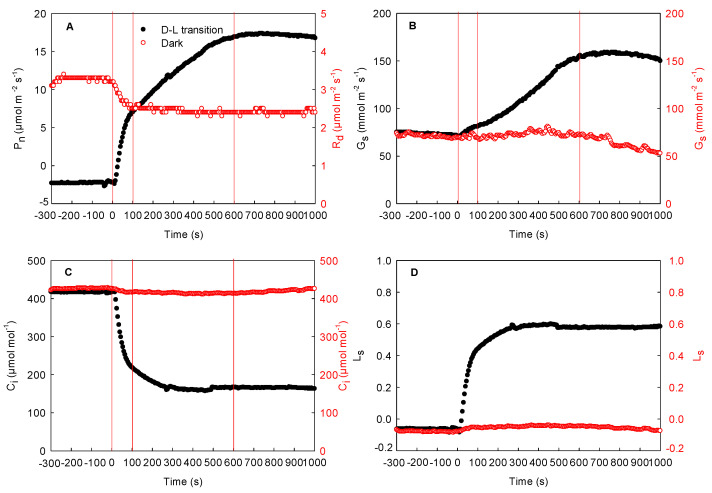
Effect of local illumination on the respiration rate of the adjacent region in the same cotton leaves. (**A**) photosynthetic induction under the dark–light transition in the local regions or respiration in darkness in the adjacent region; (**B**) stomatal conductance under the dark–light transition in the local regions or under the darkness in the adjacent region; (**C**) intercellular CO_2_ concentration under the dark–light transition in the local regions or under the darkness in the adjacent region; (**D**) stomatal limitation under the dark–light transition in the local regions or in darkness in the adjacent region. C_i_, intercellular CO_2_ concentration; D–L transition, dark–light transition; G_s_, stomatal conductance; L_s_, stomatal limitation; P_n_, photosynthetic rate; R_d_, respiration in darkness.

**Figure 5:**
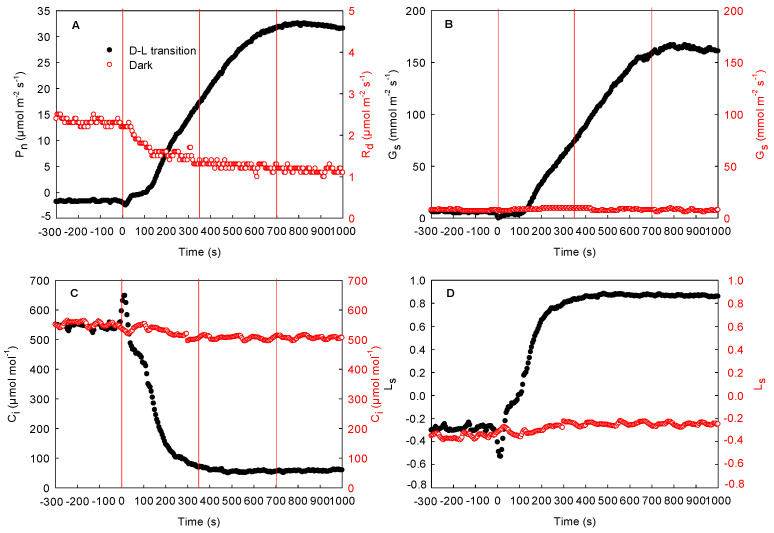
Effect of local illumination on the respiration rate of the adjacent region in the same maize leaves. (**A**) photosynthetic induction under the dark–light transition in the local regions or respiration in darkness in the adjacent region; (**B**) stomatal conductance under the dark–light transition in the local regions or under the darkness in the adjacent region; (**C**) intercellular CO_2_ concentration under the dark–light transition in the local regions or under the darkness in the adjacent region; (**D**) stomatal limitation under the dark–light transition in the local regions or in darkness in the adjacent region. C_i_, intercellular CO_2_ concentration; D–L transition, dark–light transition; G_s_, stomatal conductance; L_s_, stomatal limitation; P_n_, photosynthetic rate; R_d_, respiration in darkness.

**Figure 6:**
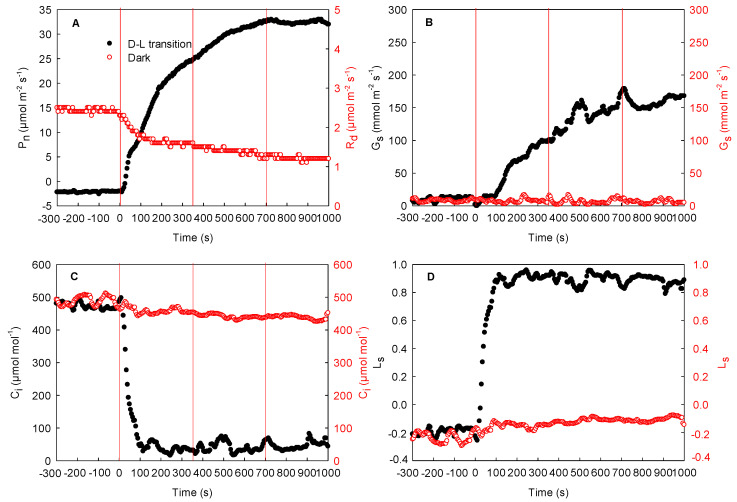
Effect of local illumination on the respiration rate of adjacent region in the same sorghum leaves. (**A**) photosynthetic induction under the dark–light transition in the local regions or respiration in darkness in the adjacent region; (**B**) stomatal conductance under the dark–light transition in the local regions or under the darkness in the adjacent region; (**C**) intercellular CO_2_ concentration under the dark–light transition in the local regions or under the darkness in the adjacent region; (**D**) stomatal limitation under the dark–light transition in the local regions or in darkness in the adjacent region. C_i_, intercellular CO_2_ concentration; D–L transition, dark–light transition; G_s_, stomatal conductance; L_s_, stomatal limitation; P_n_, photosynthetic rate; R_d_, respiration in darkness.

The effects of local illumination on the respiration rate of the adjacent unilluminated region in the cotton, maize, and sorghum leaves are shown in Figure 4, Figure 5, and Figure 6, respectively. The respiration rate was stable when the whole leaf was maintained in darkness (from −300 to 0 s). When the local region of the leaf was suddenly exposed to high light, the photosynthetic rate and stomatal conductance in this region increased gradually and then peaked, while the intercellular CO_2_ concentration decreased rapidly and then stabilized (from 0 to 1000 s). Concurrently, the stomatal limitation increased rapidly (Figure 4D and Figure 5D). During this process, respiration in the adjacent unilluminated region was reduced sharply and then stabilized. Compared with 0 s, the respiration rate of the cotton leaf dropped by about 25%, while that of the maize leaf fell by about 52% (at 1000 s). As shown in Figure 4 and Figure 5, there were no marked changes in the stomatal conductance of the adjacent unilluminated region of the leaves in the two species during the experiment, while the intercellular CO_2_ concentration declined slightly (Figure 4A–C and Figure 5A–C). Obviously, the trend of maize (C_4_ monocotyledon) leaves was similar to that of cotton ones (Figure 5). To further test these results of maize, the gas exchange in sorghum (other C_4_ monocotyledon) leaves was carefully measured under the same condition. The result of the sorghum was consistent with that of the maize (Figure 6). Consequently, illumination in local regions of the leaves resulted in a decrease in respiration rate in the adjacent region of the cotton, maize, and sorghum plants.

As local illumination decreases the respiration rate of the adjacent unilluminated region, the local illumination might also affect the photosynthetic rate in the adjacent region. Therefore, the maize plants were used as plant material for further investigation under complex light environments.

### 2.3. Effect of Local Illumination on the Photosynthetic Rate of the Adjacent Region in the Same Leaves

**Figure 7:**
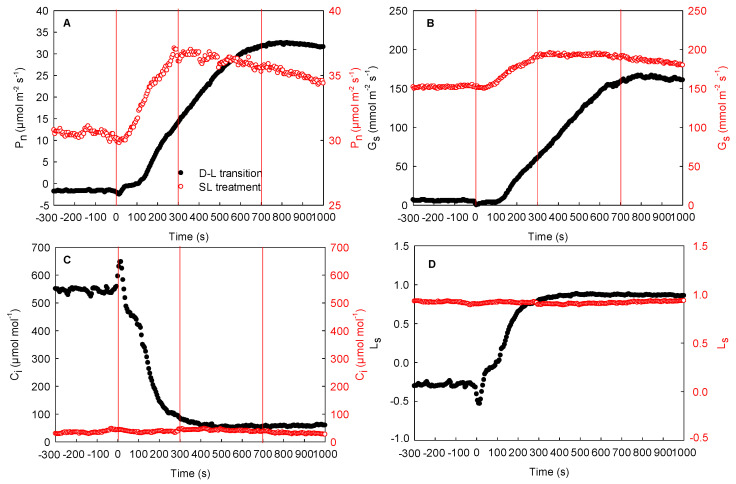
Effect of local illumination on the photosynthetic rate of the adjacent region in the same maize leaves. (**A**) photosynthetic induction under the dark–light transition in the local regions or photosynthetic rate under steady light intensity in the adjacent region; (**B**) stomatal conductance under the dark–light transition in the local regions or under steady light intensity in the adjacent region; (**C**) intercellular CO_2_ concentration under the dark–light transition in the local regions or region under steady light intensity in the adjacent; (**D**) stomatal limitation under the dark–light transition in the local regions or under steady light intensity in the adjacent region. C_i_, intercellular CO_2_ concentration; D–L transition, dark–light transition; G_s_, stomatal conductance; L_s_, stomatal limitation; P_n_, photosynthetic rate; SL treatment, steady light treatment.

Figure 7 shows the effect of local illumination on the photosynthetic rate in the adjacent region of the same maize leaves. First, when the adjacent region of the leaves was exposed to high light, the photosynthetic rate reached the maximum value and maintained a stable trend. The local regions were maintained in darkness. Thereafter, the local unilluminated regions of the leaves were suddenly exposed to high light for photosynthetic induction. During the process of photosynthetic induction in this region, the photosynthetic rate and stomatal conductance increased gradually and peaked at about 700 s (Figure 7A,B). Meanwhile, the intercellular CO_2_ concentration decreased rapidly, reaching the minimum level at 400 s and then ultimately tending to stabilize (Figure 7C). Moreover, the stomatal limitation of the maize leaves increased quickly and peaked (Figure 7D). During this process, the photosynthetic rate in the adjacent region under steady high light was also enhanced rapidly, reaching the maximum value at 300 s. Compared to 0 s, the photosynthetic rate in the adjacent illuminated region was increased by about 25% (Figure 7A). Here, the stomatal conductance trend was consistent with that of the photosynthetic rate; yet, the intercellular CO_2_ concentration did not change significantly under steady high light (Figure 7A–C). These results indicated that the photosynthetic rate of the adjacent illuminated region could be improved by the illumination of local regions in the same maize leaves.

### 2.4. Effects of the Changing Light Environment on the Respiration and Photosynthetic Rate of the Same Maize Leaves in the Field

**Figure 8:**
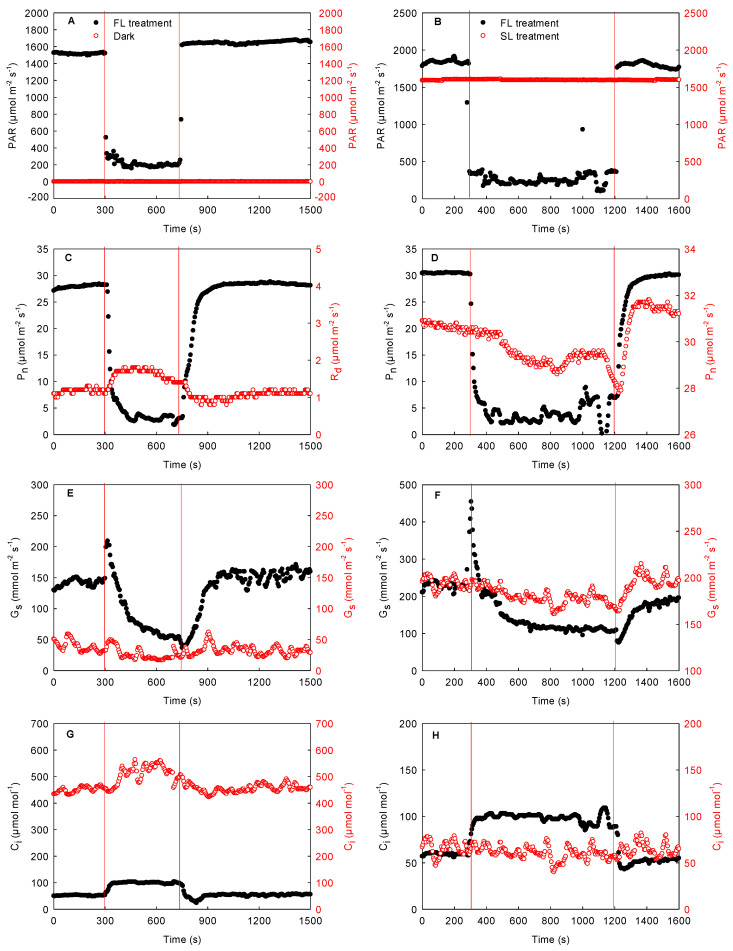
Effects of fluctuating light intensity in local regions on the respiration rate and photosynthetic rate of adjacent region in the same maize leaves in the field. (**A**) light intensity under natural fluctuating light in the local regions or in darkness in the adjacent region; (**B**) light intensity under natural fluctuating light in the local regions or under steady light intensity in the adjacent region; (**C**) photosynthetic rate under natural fluctuating light in the local regions or respiration in darkness in the adjacent region; (**D**) photosynthetic rate under natural fluctuating light in the local regions or under steady light intensity in the adjacent region; (**E**) stomatal conductance under natural fluctuating light in the local regions or in darkness in the adjacent region; (**F**) stomatal conductance under natural fluctuating light in the local regions or under steady light intensity in the adjacent region; (**G**) intercellular CO_2_ concentration under natural fluctuating light in the local regions or in darkness in the adjacent region; (**H**) intercellular CO_2_ concentration under natural fluctuating light in the local regions or under steady light intensity in the adjacent region. C_i_, intercellular CO_2_ concentration; FL treatment, fluctuating light treatment; G_s_, stomatal conductance; PAR, photosynthetically active radiation; P_n_, photosynthetic rate; R_d_, respiration in darkness; SL treatment, steady light treatment.

To further test these results, the effects of fluctuating light intensity in local regions on the respiration rate and photosynthetic rate of the adjacent region were explored in the same maize leaves in the field. As shown in Figure 8, after the photosynthetic rate stabilized in the local regions of the leaves, the decrease in the light intensity resulted in the rapid reduction in photosynthetic rate and stomatal conductance in these regions and the increase in intercellular CO_2_ concentration. During this process, the stomatal conductance in the adjacent unilluminated region dropped slightly, while the respiration rate and the intercellular CO_2_ concentration enhanced markedly. Until the light intensity of the illuminated region recovered to its original level, the photosynthetic rate and stomatal conductance increased gradually, and the intercellular CO_2_ concentration reduced. At this time, the stomatal conductance of the unilluminated region increased slightly, and both the respiration rate and the intercellular CO_2_ concentration decreased (Figure 8A,C,E,G). These results confirmed that the change in light intensity in the local regions affected the respiration rate in the adjacent unilluminated region of the same leaves in the field.

Furthermore, in the field, we also measured the effect of changes in local light intensity on the photosynthetic rate of the adjacent region in the same maize leaves under steady high light. After the photosynthetic rate stabilized in the local regions, the decrease in the natural light intensity of the local regions resulted in a rapid decrease in photosynthetic rate and stomatal conductance and an increase in intercellular CO_2_ concentration (Figure 8B,D,F,H). Meanwhile, the photosynthetic rate under steady light intensity in the adjacent region also showed a decreasing trend, while the stomatal conductance declined slightly, and the intercellular CO_2_ concentration did not change significantly (Figure 8B,D,F,H). Later, the photosynthetic rate and stomatal conductance increased rapidly after the light intensity of the local regions recovered to its original level, while the intercellular CO_2_ concentration dropped. Concurrently, the photosynthetic rate under steady light intensity in the adjacent region also increased significantly, and the stomatal conductance increased slightly. There was no striking change in intercellular CO_2_ concentration in the adjacent region (Figure 8B,D,F,H). These data suggested that the changes in local light intensity affected the photosynthetic rate of the adjacent region in the same leaves.

## 3. Discussion

### 3.1. Lateral CO_2_ Diffusion Supports Photosynthesis in Maize Leaves

Previous studies have shown that the lateral gas phase diffusion of CO_2_ takes place in C_3_ plants (both heterobaric and homobaric leaves) and supports photosynthesis in adjacent regions [10,11,12]. In this study, a stable respiration rate was reached (about 3.2 μmol m^−2^ s^−1^) when entire cotton leaves were maintained in darkness (Figure 4A). After local regions of the leaves were subjected to high light, the photosynthetic initiation of these regions increased the utilization of CO_2_. At the initial stage of photosynthetic induction of the local regions, the stomatal conductance in these regions of the cotton leaves increased very slowly. At this time, CO_2_ cannot be quickly taken up from the atmosphere through the stomata, and thus, the intercellular CO_2_ concentration decreased rapidly. More importantly, the respiration rates of the adjacent unilluminated region of the cotton leaves decreased rapidly, as did the intercellular CO_2_ concentration (Figure 4). These results are consistent with previous studies on C_3_ plants [24]. Accordingly, the CO_2_ produced via respiration in the adjacent unilluminated region was laterally transported to the local illuminated regions by the gas phase pathway and utilized in photosynthesis. In this study, we found that maize and sorghum also exhibited the same trend as cotton leaves (Figure 4, Figure 5 and Figure 6). Therefore, we deduced that in maize and sorghum leaves, the CO_2_ produced via respiration of the adjacent unilluminated region could also be transported laterally by the gas phase pathway to the neighboring illuminated regions.

Further analysis showed that the adjacent region of the maize leaves had a stable photosynthetic rate when they were maintained under steady high light, while the local regions exhibited respiration before illumination. Thereafter, the local unilluminated regions were suddenly exposed to high light for photosynthesis induction. Due to the slow increase in stomatal conductance at the initial stage of photosynthesis induction, these regions could not quickly take up CO_2_ from the ambient air through the stomata, resulting in a rapid decline in intercellular CO_2_ concentration. Concurrently, the CO_2_ produced by self-respiration in these regions may be primarily used for photosynthesis. Alternatively, the stomatal conductance of the adjacent illuminated region increased considerably without an increase in light intensity, and the photosynthetic rate also increased rapidly from about 30 to 37 μmol m^−2^ s^−1^ (Figure 7), indicating that the initiation of photosynthesis induction in the local regions of the same leaf enhanced the photosynthetic rate in the adjacent illuminated region under steady high light. In addition to maize, sorghum leaves also showed similar phenomena (data not shown). Therefore, these findings further confirm that lateral CO_2_ diffusion may also occur in C_4_ plants, such as maize and sorghum, supporting photosynthesis in the adjacent illuminated region of the same leaves.

### 3.2. Mechanism of Lateral CO_2_ Diffusion inside the Maize Leaves

**Figure 9:**
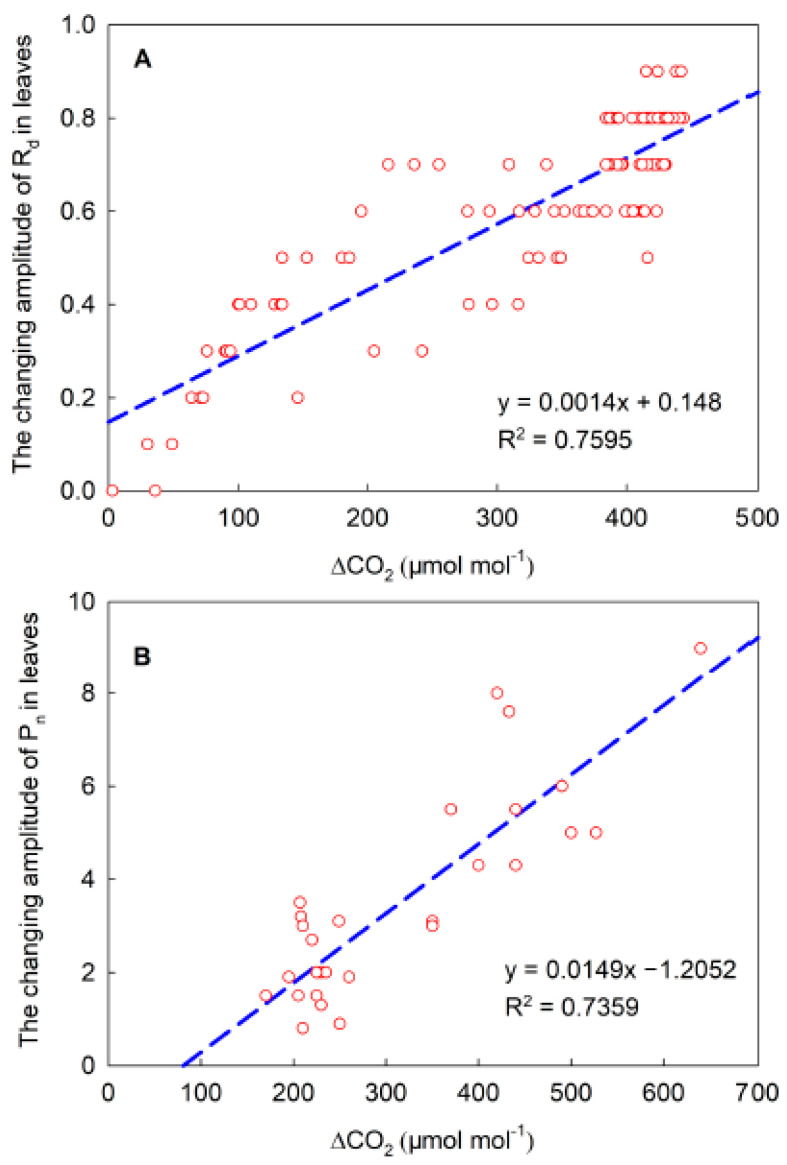
Linear correlation between ΔCO_2_ (CO_2_ pressure difference) and the changing amplitude of lateral CO_2_ diffusion. (**A**) R_d_ of maize and sorghum leaves; (**B**) P_n_ of various plant species, including maize, sorghum, cotton, sunflower, cucumber, soybean, and spinach.

Many studies have shown that the lateral diffusion of CO_2_ is related to mesophyll porosity. Large mesophyll cell porosity is conducive to CO_2_ diffusion [11,12,25]. In our investigation, however, the porosities of maize and sorghum were about twice as low as those of cotton and cucumber (Figure 2). It has previously been shown that when gas exchange is measured using double-gasket clamp-on leaf chambers, the enhancement of the local CO_2_ concentration of the leaves (outer leaf chamber) increases the respiration rate of the adjacent region (inner leaf chamber), proving that the ΔCO_2_ between the inner and outer leaf chambers leads to the lateral CO_2_ diffusion between the adjacent regions inside the leaf [10,24]. At the beginning of the experiment, local regions of the maize leaves showed a stable respiration in darkness, while the adjacent region maintained a steady photosynthesis under steady high light (Figure 7). At the same time, the CO_2_ concentration of the intercellular airspace in the two regions (the local darkened region and the adjacent illuminated region) was about 500 and 60 μmol mol^−1^, respectively. The former was about eight times higher than that of the latter. Under this condition, the high ΔCO_2_ was accompanied by obvious lateral CO_2_ diffusion, which was reflected by the clear changes in photosynthetic rate in the adjacent illuminated region. Furthermore, when the local unilluminated regions were quickly exposed to high light, the stomatal conductance in these regions was only about 10 mmol m^−2^ s^−1^ at the initial stage of photosynthesis induction, and so, the intercellular CO_2_ concentration reduced sharply, which resulted in a rapid decrease in the ΔCO_2_ between the two regions inside the leaf (Figure 7). With the decrease in ΔCO_2_, the lateral CO_2_ diffusion between adjacent regions also dropped, which was revealed by no further increase in photosynthetic rate. Under field conditions, when the light intensity in local regions of the leaves decreased, the photosynthetic rate declined quickly and the intercellular CO_2_ concentration enhanced rapidly, whereas there were no remarkable changes in the intercellular CO_2_ concentration of the adjacent region exposed to constant light intensity. Consequently, the enhanced ΔCO_2_ between the adjacent regions may result in the diffusion of CO_2_ from the local regions under weak light to the adjacent region under constant high light, as demonstrated by the decrease in photosynthetic rate (Figure 8). Similarly, under field conditions, the increase in the light intensity of the local regions in the leaves resulted in an increase in photosynthetic rate, which further caused a reduction in the ΔCO_2_ between the adjacent regions and increased the steady photosynthetic rate in the adjacent region (Figure 8). Thus, the lateral CO_2_ diffusion from the original weak light regions to the high-light region decreased. As the changing and patchy illumination induced the enhancement of the ΔCO_2_ between adjacent regions of the same leaves, it was likely that the lateral CO_2_ diffusion was mainly related to the pressure difference between the adjacent regions in the same leaf. We noticed that when the ΔCO_2_ between the adjacent regions of the maize leaves was about eight times higher, the change in photosynthetic rate reached approximately 8 μmol m^−2^ s^−1^, whereas when the ΔCO_2_ between the adjacent regions was about twice as high, the change in photosynthetic rate was about 2 μmol m^−2^ s^−1^, thus confirming that lateral CO_2_ diffusion in maize leaves is related to the ΔCO_2_. Additionally, our results also showed that the ΔCO_2_ between the adjacent regions of the same leaf was linearly correlated with the changing amplitude of lateral CO_2_ diffusion in maize, sorghum, and other species (Figure 9). Accordingly, we thought that the lateral CO_2_ diffusion between the adjacent regions is well reflected by the formula of current (I = U/R). When the diffusion resistance of CO_2_ in the leaves remains constant, the larger pressure difference between the adjacent regions of the same leaf is conducive to lateral CO_2_ diffusion. In the converse scenario, lateral CO_2_ diffusion is restricted.

In a previous study, the ΔCO_2_ between neighboring regions inside maize leaves was found to be relatively small, which may have resulted in little lateral diffusion inside the maize leaves by the gas phase pathway [19,20,26]. In our study, the larger changes in steady photosynthetic rate demonstrated that the extent of CO_2_ lateral diffusion in maize was significant (Figure 6, Figure 7 and Figure 8). However, due to the low porosity, long lateral path lengthening, and low conductance of the intercellular airspace in the maize leaves (Figure 1 and Figure 2), it was, therefore, possible that lateral CO_2_ diffusion might be not completely dependent on gaseous diffusion through the intercellular space. Carbonic anhydrase (CA) plays an important role in the initial fixation of CO_2_ [27,28,29,30,31]. More importantly, the CA activity in the mesophyll cytoplasm of C_4_ plants is higher than that of C_3_ plants, while CA in the leaves of C_3_ plants is mainly distributed in the chloroplasts [32,33,34,35]. The content of CA in the cytoplasm is low. Accordingly, the CO_2_ produced during respiration in the leaves of C_4_ plants (such as maize and sorghum) might also diffuse partially through the liquid-phase pathway. It is likely that both the gas- and liquid-phase pathways may co-influence the lateral CO_2_ diffusion between different regions in the same maize leaf, further influencing leaf photosynthesis.

### 3.3. The Significance of Lateral CO_2_ Diffusion within the Leaf

**Figure 10:**
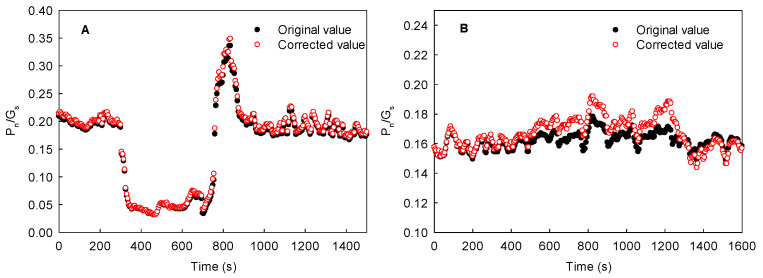
Effects of a changing light environment on the WUE (P_n_/G_s_) of local (**A**) and adjacent regions (**B**) in the same maize leaves in the field. Data from Figure 8. WUE, water use efficiency; corrected value, (P_n–F_ or P_n–S_ + ΔP_n_)/G_s_; P_n–F_, photosynthetic induction under fluctuating light; P_n–S_, photosynthesis under steady light intensity; ΔP_n_, the changes in P_n–S_ of adjacent region during the transition of the local regions from dark or low-light regions to high-light regions in the same leaves.

More than 10 plant species, including maize, sorghum, cotton, sunflower, soybean, cucumber, and spinach, were analyzed in the present study (Figure 9). We found that the lateral diffusion of CO_2_ produced by leaf respiration not only reduced the value of respiration rate (about 50%), but also resulted in the underestimation of steady photosynthetic rate (by about 20%). Theoretically, the more drastic the changes in light intensity that take place under a patchy light environment at the same time between neighboring regions of the same leaf, the more obvious the underestimation of steady photosynthetic rate. Thus, lateral CO_2_ diffusion into the leaves under a heterogenous light environment may cause a striking underestimation of steady photosynthetic rate and carbon assimilation by gas exchange measurements. In the field, the lateral CO_2_ diffusion caused by a complex or patchy light environment also resulted in the fluctuation or instability of photosynthesis when the partial leaf region was measured under steady light intensity, which may further lead to considerable differences in the photosynthetic rate measured in different studies. Moreover, the underestimation of photosynthetic rate due to the lateral CO_2_ diffusion from dark or low-light regions to high-light regions also resulted in the undervaluation of the water use efficiency (WUE) to a certain extent (Figure 10). Therefore, the lateral diffusion of CO_2_ in the leaves under a complex light environment is also beneficial for improving WUE.

## 4. Materials and Methods

### 4.1. Plant Materials and Experimental Design

Experiments were carried out in 2021 at the Institute of Botany at the Chinese Academy of Sciences in Beijing. C_4_ plants (maize and sorghum) and C_3_ plants (cotton, cucumber, sunflower, soybean, and spinach) were used as the experimental materials. Before the experiment, the maize, sorghum, and cotton seeds were imbibed on wet culture dishes for 48 h in the dark at 25 °C. Germinated seeds were then planted in containers (21 cm in diameter and 30 cm in height) that had seepage pores and were filled with a 1:1 mixture of loess and peat. Potted seedlings were cultured in the field. Normal water and fertilizer management was performed throughout to avoid potential nutrient and drought stresses. In addition, other species (cucumber, sunflower, soybean, and spinach) came from the nursery of China National Botanical Garden. When the plant grew 7–8 leaves, gas exchange due to respiration and photosynthesis under controlled light and natural light was measured using only fully expanded leaves.

### 4.2. Determination of Anatomical Structure and Leaf Porosity

Semi-thin leaf sections were observed with reference to a method described by Gong et al. [36] and Jiang et al. [37]. Leaf segments (2 mm × 3 mm) without major veins were cut from the intermediate part of the leaf with a razor blade at 5:00. The segments were fixed in 3% glutaraldehyde, which was formulated with 8% glutaraldehyde, 0.1 mol L^–1^ phosphate buffer, and distilled water, at 4 °C. Each segment was pumped by vacuum for complete immersion. During sample preparation, the segment was rinsed with phosphate-buffered solution three times, fixed in 1% osmic acid solution for 6 h, rinsed again three times with 0.1 mol L^–1^ phosphate buffer, and then dehydrated in a graded series of ethanol and acetone solutions and embedded in Spurr’s resin (Ladd Research Industries, Williston, VT, USA). Light microscopy was carried out with 1 μm thick transverse sections of the leaf cut with a glass knife on an ultramicrotome (Leica Ultracut R, Leica Microsystems, Wetzlar City, Germany) and stained with 0.5% toluidine blue. Leaf structure was observed and photographed with a light microscope (Nikon-E800, Scientific Imaging Inc., Salt Lake City, UT, USA). Data analysis was conducted in Adobe Acrobat 7.0 Professional.

Mesophyll porosity (θ_ias_), tortuosity, lateral diffusivity, and IAS conductance were determined as described by Syvertsen et al. [14] and Earles et al. [7]. Briefly, the fraction of mesophyll volume occupied by the IAS was determined as

θ_ias_ = 1 − A_m_/(WL_mes_) (1)

where A_m_, W, and L_mes_ are the total cross-sectional region of the mesophyll cells, width of the section, and mesophyll thickness between the two epidermises, respectively.

The tortuosity factor, τ (m^2^ m^−2^), was defined as the ratio of the diffusive path length within the IAS (i.e., the actual path from the stomata to a cell surface; geodesic distance (L_geo_)) to the straight path length in the absence of any physical obstacles to diffusion between the stomata and the cell surface (Euclidean distance, L_Euc_). To calculate L_geo_ and L_Euc_, we first generated a binary image of the stomatal inlets for each leaf. Then, we calculated two geodesic distance maps and Euclidean distance maps using Adobe Acrobat 7.0 Professional and then calculated τ at all voxels along the mesophyll surface as

τ = L^2^_geo_/L^2^_Euc_ (2)

To calculate lateral path lengthening, λ (m m^−1^), we used the previously generated distance map, L_Euc_. Then, we created a second distance map, again using the Adobe Acrobat 7.0 Professional, to quantify the shortest unobstructed straight-line distance between the lower epidermis and all points along the mesophyll surface, L_epi_. From these two distance maps, we calculated λ at all voxels along the mesophyll surface as:

λ = L_Euc_/L_epi_ (3)

Using tortuosity and lateral diffusivity factors, we then calculated leaf-level IAS conductance as:

g_ias_ = (θ_ias_D_m_)/(0.5 L_mes_τλ) (4)

where D_m_ is the diffusivity of CO_2_ in air (m^2^ s^−1^).

### 4.3. Determination of Gas Exchange under a Controlled Light Environment

As shown in Figure 3, photosynthetic induction under the dark–light transition, the photosynthetic rate in steady light intensity, and respiration in darkness (R_d_) under a controlled light environment were measured with an open gas exchange system (Ciras-2, PP Systems, Amesbury, MA, USA) between 08:00 and 12:00 on a sunny day. During this process, CO_2_, gas flow, and humidity were maintained at 380 ± 20 μmol mol^−1^, 200 mL min^−1^, and 75% ± 5%, respectively, with the ambient temperature maintained in the leaf chamber. The light intensity was controlled at 0 or 1600 µmol m^−2^ s^−1^. Data were recorded every 5 s. The experiment under a controlled light environment was conducted in the greenhouse.

(1) First, the measurement of the photosynthetic induction under the dark–light transition in the local regions was carried out. In this process, the light intensity in the local regions was maintained in darkness (0 μmol^−2^ m^−2^ s^–1^). After stabilization of the respiration rate in the local regions, the Light-Emitting Diode (LED) of the leaf chamber in the local regions was then turned on (light intensity: 1600 μmol m^−2^ s^−1^). During this process, the photosynthetic induction under the dark–light transition was recorded every 5 s (Figure 3A).
(2) Secondly, the measurement of respiration in darkness and photosynthetic rate under steady light intensity in the adjacent region was carried out: The light intensity in the adjacent region was controlled to 0 or 1600 μmol m^−2^ s^−1^ via an LED of the leaf chamber, while the local regions were maintained in darkness. After a stable respiration rate or a steady-state photosynthetic rate in the adjacent region was achieved, the local regions were subjected to a light intensity of 1600 μmol m^−2^ s^−1^ controlled by an LED, and the changes in respiration rate or photosynthetic rate in the adjacent region under steady light intensity were recorded every 5 s (Figure 3B).

### 4.4. Determination of Gas Exchange in the Field

As shown in Figure 3, photosynthesis induction in fluctuating light, the photosynthetic rate in steady light intensity, and respiration in darkness were measured with an open gas exchange system, Ciras-2 (PP Systems, Amesbury, MA, USA), between 08:00 and 12:00 on a sunny day. During this process, CO_2_, gas flow, and humidity were maintained at 380 ± 20 μmol mol^−1^, 200 mL min^−1^, and 75% ± 5%, respectively, with the ambient temperature maintained in the leaf chamber. A timed program was used to record a point every 5 s. The experiment was conducted in the field.

(1) To investigate the effects of natural fluctuating light on photosynthesis in the local regions, the measurements in the local regions were carried out in the field with a gas exchange system (removal of LED light source) (Figure 3A).
(2) To study the effects of natural fluctuating light on respiration or photosynthetic rate in the adjacent region, the measurements in the adjacent region were performed with a gas exchange system (with LED light source). The light intensity of the leaf chamber (adjacent region) was controlled to 0 or 1600 µmol m^−2^ s^−1^, and the respiration rate or photosynthetic rate in steady light intensity was determined, respectively (Figure 3B).

### 4.5. Statistical Analysis

Data were analyzed using one-way analysis of variance (ANOVA) and compared with the significant difference (LSD) multiple comparison test using SPSS (version 25, IBM Corp., Armonk, NY, USA). Statistical significance was determined at *p* < 0.05. The graphics software SigmaPlot (version 12.5) was used to create illustrations.

## 5. Conclusions

Accordingly, we thought that increasing the CO_2_ pressure difference between the adjacent regions inside the maize leaves may result in the lateral diffusion of CO_2_ and support photosynthesis in the adjacent region to a certain extent. Under complex light environments, lateral CO_2_ diffusion inside the leaves is of great significance for maintaining carbon assimilation and improving WUE.

## Data Availability

Not applicable.

## References

[1] von Caemmerer S., Farquhar G.D. (1981). Some relationships between the biochemistry of photosynthesis and the gas exchange of leaves. Planta.

[2] Evans J.R., Kaldenhoff R., Genty B., Terashima I. (2009). Resistances along the CO_2_ diffusion pathway inside leaves. J. Exp. Bot..

[3] Evans J.R. (2020). Mesophyll conductance: Walls, membranes and spatial complexity. New Phytol..

[4] Berghuijs H.N.C., Yin X.Y., Ho Q.T., Retta M.A., Nicolai B.M., Struik P.C. (2019). Using a reaction-diffusion model to estimate day respiration and reassimilation of (photo)respired CO_2_ in leaves. New Phytol..

[5] Ye M., Zhang Z.C., Huang G.J., Li Y. (2022). Leaf photosynthesis and its temperature response are different between growth stages and N supplies in rice plants. Int. J. Mol. Sci..

[6] Ligrone R., Duckett J.G., Renzaglia K.S. (2012). Major transitions in the evolution of early land plants: A bryological perspective. Ann. Bot..

[7] Earles J.M., Theroux-Rancourt G., Roddy A.B., Gilbert M.E., McElrone A.J., Brodersen C.R. (2018). Beyond porosity: 3D leaf intercellular airspace traits that impact mesophyll conductance. Plant Physiol..

[8] Barbosa M.A.M., Chitwood D.H., Azevedo A.A., Araújo W.L., Ribeiro D.M., Peres L.E.P., Martins S.C.U., Zsögön A. (2019). Bundle sheath extensions affect leaf structural and physiological plasticity in response to irradiance. Plant Cell Environ..

[9] Parkhurst D.F. (1994). Diffusion of CO_2_ and other gases inside leaves. New Phytol..

[10] Jahnke S., Krewitt M. (2002). Atmospheric CO_2_ concentration may directly affect leaf respiration measurement in tobacco, but not respiration itself. Plant Cell Environ..

[11] Pieruschka R., Schurr U., Jahnke S. (2005). Lateral gas diffusion inside leaves. J. Exp. Bot..

[12] Pieruschka R., Chavarria-Krauser A., Schurr U., Jahnke S. (2010). Photosynthesis in lightfleck areas of homobaric and heterobaric leaves. J. Exp. Bot..

[13] Sáez P.L., Bravo L.A., Cavieres L.A., Vallejos V., Sanhueza C., Font-Carrascosa M., Gil-Pelegrín E., Peguero-Pina J.J., Galmés J. (2017). Photosynthetic limitations in two Antarctic vascular plants: Importance of leaf anatomical traits and Rubisco kinetic parameters. J. Exp. Bot..

[14] Syvertsen J.P., Lloyd J., McConchie C., Kriedemann P.E., Farquhar G.D. (1995). On the relationship between leaf anatomy and CO_2_ diffusion through the mesophyll of hypostomatous leaves. Plant Cell Environ..

[15] Niinemets Ü., Reichstein M. (2003). Controls on the emission of plant volatiles through stomata: Differential sensitivity of emission rates to stomatal closure explained. J. Geophys. Res..

[16] Tomás M., Flexas J., Copolovici L., Galmés J., Hallik L., Medrano H., Ribas-Carbó M., Tosens T., Vislap V., Niinemets Ü. (2013). Importance of leaf anatomy in determining mesophyll diffusion conductance to CO_2_ across species: Quantitative limitations and scaling up by models. J. Exp. Bot..

[17] Harwood R., Théroux-Rancourt G., Barbour M.M. (2021). Understanding airspace in leaves: 3D anatomy and directional tortuosity. Plant Cell Environ..

[18] Lawson T., Morison J. (2006). Visualising patterns of CO_2_ diffusion in leaves. New Phytol..

[19] Morison J.I.L., Lawson T. (2007). Does lateral gas diffusion in leaves matter?. Plant Cell Environ..

[20] Morison J.I.L., Lawson T., Cornic G. (2007). Lateral CO_2_ diffusion inside dicotyledonous leaves can be substantial: Quantification in different light intensities. Plant Physiol..

[21] Long S.P., Farage P.K., Bolha´r-Nordenkampf H.R., Rohrhofer U. (1989). Separating the contribution of the upper and the lower mesophyll to photosynthesis in *Zea mays* L. leaves. Planta.

[22] Graham E.A., Mulkey S.S., Kitajima K., Phillips N.G., Wright S.J. (2003). Cloud cover limits net CO_2_ uptake and growth of a rainforest tree during tropical rainy seasons. Proc. Natl. Acad. Sci. USA.

[23] Xue W., Lindner S., Dubbert M., Otieno D., Ko J.H., Muraoka H., Werner C., Tenhunen J. (2017). Supplement understanding of the relative importance of biophysical factors in determination of photosynthetic capacity and photosynthetic productivity in rice ecosystems. Agric. Forest Meteorol..

[24] Pieruschka R., Schurr U., Jensen M., Wolff W.F., Jahnke S. (2006). Lateral diffusion of CO_2_ from shaded to illuminated leaf parts affects photosynthesis inside homobaric leaves. New Phytol..

[25] Pieruschka R., Chavarria-Krauser A., Cloos K., Scharr H., Schurr U., Jahnke S. (2008). Photosynthesis can be enhanced by lateral CO_2_ diffusion inside leaves over distances of several millimeters. New Phytol..

[26] Morison J.I.L., Gallouet E., Lawson T., Cornic G., Herbin R., Baker N.R. (2005). Lateral diffusion of CO_2_ in leaves is not sufficient to support photosynthesis. Plant Physiol..

[27] Hatch M.D., Burnell J.N. (1990). Carbonic anhydrase activity in leaves and its role in the first step of C_4_ photosynthesis. Plant Physiol..

[28] Badger M.R., Price G.D. (1994). The role of carbonic anhydrase in photosynthesis. Annu. Rev. Plant Phys..

[29] Poschenrieder C., Fernández J.A., Rubio L., Pérez L., Terés J., Barceló J. (2018). Transport and use of bicarbonate in plants: Current knowledge and challenges ahead. Int. J. Mol. Sci..

[30] Wang L.L., Liang J.J., Zhou Y., Tian T., Zhang B.L., Duanmu D.Q. (2021). Molecular characterization of carbonic anhydrase genes in *Lotus japonicus* and their potential roles in symbiotic nitrogen fixation. Int. J. Mol. Sci..

[31] Chatterjee J., Coe R.A., Acebron K., Thakur V., Yennamalli R.M., Danila F., Lin H.C., Balahadia C.P., Bagunu E., Padhmapriya P.O.S. (2021). A low CO_2_-responsive mutant of Setaria viridis reveals that reduced carbonic anhydrase limits C_4_ photosynthesis. J. Exp. Bot..

[32] Poincelot R.P. (1972). Intracellular distribution of carbonic anhydrase in spinach leaves. Biochim. Et Biophys. Acta (BBA)-Enzymol..

[33] Friso G., Majeran W., Huang M., Sun Q., van Wijk K.J. (2010). Reconstruction of metabolic pathways, protein expression, and homeostasis machineries across maize bundle sheath and mesophyll chloroplasts: Large-scale quantitative proteomics using the first maize genome assembly. Plant Physiol..

[34] Bräutigam A., Mullick T., Schliesky S., Weber A.P.M. (2011). Critical assessment of assembly strategies for non-model species mRNA-Seq data and application of next-generation sequencing to the comparison of C_3_ and C_4_ species. J. Exp. Bot..

[35] Studer A.J., Gandin A., Kolbe A.R., Wang L., Cousins A.B., Brutnell T.P. (2014). A limited role for carbonic anhydrase in C_4_ photosynthesis as revealed by a *ca1ca2* double mutant in maize. Plant Physiol..

[36] Gong Y., Chen H.M., Jiang C.D., Shi L. (2014). Quantification of leaf anatomical structure and its application in a C_4_ plant, sorghum. Chin. Bull. Bot..

[37] Jiang C.D., Wang X., Gao H.Y., Shi L., Chow W.S. (2011). Systemic regulation of leaf anatomical structure, photosynthetic performance, and high-light tolerance in sorghum. Plant Physiol..

